# A Novel Approach to Reducing Chemoresistance in Advanced Ovarian Cancer: The Effect of Itraconazole—A Single-Institution Randomized Placebo-Controlled Trial

**DOI:** 10.3390/curroncol33010021

**Published:** 2025-12-31

**Authors:** Ahmed E. S. Besheir, Sahar M. El-Hagar, Hesham A. Tawfik, Tarek M. Mostafa

**Affiliations:** 1 Clinical Pharmacy Department, Faculty of Pharmacy, Tanta University, Tanta 31511, Egypt; sahr.elhaggar@pharm.tanta.edu.eg (S.M.E.-H.); tarek.mostafa@pharm.tanta.edu.eg (T.M.M.); 2 Clinical Oncology and Nuclear Medicine Department, Faculty of Medicine, Tanta University, Tanta 31511, Egypt; hesham.gabr@med.tanta.edu.eg

**Keywords:** ovarian cancer, itraconazole, tumor response, QOL, chemotherapy

## Abstract

**Background:** The five-year survival rate of patients with ovarian cancer remains less than 50%, secondary to chemotherapy resistance. **Purpose:** This study aims to evaluate the effects of itraconazole as a supplementary treatment with paclitaxel and carboplatin on malignancy response and in preventing the initial development of chemoresistance in chemotherapy-naïve patients with advanced ovarian epithelial cancer. **Method:** This randomized placebo-controlled double-blind study involved 60 chemotherapy-naïve patients with advanced epithelial ovarian malignancy who were randomized into two arms; the placebo and itraconazole groups. The placebo group received six chemotherapy cycles and four inactive capsules, while the itraconazole group received six chemotherapy cycles and 400 mg oral itraconazole for five days per cycle. **Results:** Following completion of six chemotherapy cycles and when contrasted with the control arm, the itraconazole arm demonstrated statistically significant improvements in tumor response. The objective response rate was 80% in the itraconazole group compared with 47% in the placebo group (*p* = 0.015), while the disease control rate was 100% versus 80%, respectively (*p* = 0.023). The median progression-free survival (PFS), defined as the time point at which 50% of patients experienced disease progression or death, was 13.5 months for the overall study population. PFS was evaluated as a fixed-time endpoint at 18 months following completion of chemotherapy for the overall study population. Progression-free survival was significantly improved in the itraconazole group, with 70% of patients remaining progression-free compared with 26.7% in the placebo group (*p* = 0.001). Also, the itraconazole group produced significant declines in the serum levels of CA-125 (*p* = 0.005) and p-glycoprotein (*p* = 0.042) with significant elevation in VEGFR-2 (*p* = 0.006) as compared to the control group. Itraconazole was safe and its use was associated with a significant improvement in the quality of life (QOL). **Conclusions:** Itraconazole could represent a promising add-on therapy to enhance tumor response to chemotherapy in patients with ovarian cancer.

## Simple Summary

This randomized, placebo-controlled double-blind clinical trial evaluated itraconazole as an adjunct to standard paclitaxel–carboplatin chemotherapy in 60 patients with advanced epithelial ovarian cancer. Participants were divided into two groups: one received standard chemotherapy plus placebo, and the other received chemotherapy plus 400 mg itraconazole for five days per cycle. The itraconazole group showed significantly improved overall and disease control response rates, progression-free survival, and a reduction in serum CA-125 and P-glycoprotein levels, with increased VEGFR-2 levels as compared to the control group. The quality of life was markedly improved across physical, emotional, and social domains, with fewer chemotherapy-related side effects. Importantly, itraconazole was well-tolerated and did not increase hematologic or systemic toxicities. These findings suggest that itraconazole may enhance tumor response, mitigate chemoresistance, and improve quality of life in patients with advanced ovarian cancer, supporting its potential as a safe, cost-effective adjunctive therapy; however, these findings require further validation.

## 1. Introduction

Among gynecological malignancies, ovarian cancer ranks as one of three conditions associated with a great fatality rate. Clinical manifestations of ovarian cancer are usually non-specific, and the effective screening methodologies for this cancer remain unavailable. Consequently, the majority of individuals with ovarian cancer are diagnosed during the late-stage disease, resulting in a five-year overall survival rate of merely 47% [1]. This malignancy demonstrates elevated relapse frequency, suboptimal detection rates, and unfavorable clinical outcomes, which seriously threatens women’s life and health [2].

Currently, the primary therapeutic approaches for this malignancy include debulking surgical procedures, platinum-containing chemotherapeutic regimens, and subsequent maintenance treatment utilizing bevacizumab alongside poly ADP-ribose polymerase (PARP) inhibitors [3]. Nevertheless, approximately 65–80% of individuals diagnosed with late-stage ovarian malignancy develop relapse [4]. Ultimately, as platinum-based chemotherapy treatment cycles progress, the malignancy develops platinum resistance. This phenomenon represents the primary factor contributing to unfavorable clinical outcomes among individuals with late-stage ovarian cancer [5]. Consequently, enhancing survival outcomes and clinical trajectories for individuals with ovarian malignancy represents a critical therapeutic priority in clinical settings [6].

Itraconazole has demonstrated antineoplastic properties by promoting cell cycle disruption, programmed cell death, and autophagy, and by concurrently targeting numerous oncogenic pathways to suppress cancer cell proliferation, metastasis, and invasive characteristics. Chemotherapeutic resistance development among cancer patients constitutes a significant prognostic determinant. Itraconazole was reported to function as an established p-glycoprotein antagonist capable of overcoming multidrug resistance mechanisms [7]. Furthermore, its potent antineoplastic properties and its favorable safety profile make itraconazole an attractive candidate for multiple clinical investigations, which demonstrated substantial therapeutic efficacy. Within a primary xenograft model which implicated human non-small cell lung cancer (NSCLC), itraconazole augmented the effectiveness of cisplatin chemotherapy [8].

A previous study examined the growth-inhibitory characteristics of itraconazole through the implication of ovarian epithelial cancer (EOC) cell lines (SKOV3ip1) and vascular endothelial cell lines (HUVEC and SVEC4-10), and investigated angiogenic factor (VEGFR2, p-ERK, p-PLCr1/2), hedgehog (Gli1, Ptch1, SMO), and mTOR (pS6K1) signaling pathways to clarify itraconazole-related mechanistic action. Furthermore, investigators evaluated the synergistic effects of itraconazole and paclitaxel via orthotopic mouse models, utilizing established EOC cells (SKOV3ip1 or HeyA8) together with patient-derived xenografts (PDXs). Itraconazole administration suppressed endothelial cell proliferation in concentration-dependent fashion. Moreover, the antiproliferative activity of itraconazole was correlated with suppression of hedgehog, mTOR signaling cascades, and angiogenesis. Within EOC xenograft models employing SKOV3ip1or HeyA8, animals that received combined itraconazole and paclitaxel therapy demonstrated substantially reduced tumor mass as compared to the control, paclitaxel monotherapy, or itraconazole monotherapy groups [9].

Therefore, and based on the aforementioned information, this investigation sought to assess the impact of itraconazole as adjunctive treatment to paclitaxel and carboplatin on malignancy response and in preventing th initial development of chemoresistance in chemotherapy-naïve patients with advanced ovarian epithelial cancer.

## 2. Patients and Methods

In this randomized, placebo-controlled, double-blind parallel clinical trial, a total number of 60 women with advanced epithelial ovarian malignancy (stages III and IV) who were chemotherapy-naïve patients was recruited from Tanta University Hospital, Oncology Department, Tanta, Egypt between November 2022 and October 2024, and were enrolled and successfully completed the entire course of the study. The research procedures adhered to the Helsinki Declaration’s ethical principles established in 1964. Institutional authorization was obtained from the Tanta University Research Ethics Committee, Tanta, Egypt (Approval reference 35929/10/22). The study was registered on Clinicaltrials.gov (Identifier: NCT05591560) on 17 October 2022. Informed written consents were obtained from all participants.

Patients were randomly allocated into two arms: the control arm (n = 30) which received six chemotherapy cycles (21-day intervals) which consisted of premedication therapy (granisetron 1 mg IV, dexamethasone 10 mg IV, diphenhydramine 25 mg IV and famotidine 20 mg IV in 100 mL normal saline over 15 min) followed by paclitaxel (60 mg/m^2^) intravenously over one hour, then carboplatin area under curve 2 (AUC 2) intravenously over 30 min on days 1, 8, and 15. Additionally, the four placebo capsules were administered over five days (two days pre-chemotherapy, chemotherapy day, and two days post-chemotherapy) and the experimental arm (Itraconazole group; n = 30) received identical chemotherapeutic protocol plus 400 mg of oral itraconazole (four capsules, 100 mg each, Itrapex^®^, Apex Pharmaceuticals Industry, Badr city, Egypt) over five days, with “two days pre-chemotherapy, chemotherapy day, and two days post-chemotherapy”. Patients in both study arms did not receive maintenance bevacizumab or PARP inhibitor therapies at any point during the study period [10].

Patients were randomized in 1:1 ratio into the two study groups using computer-generated random number tables and the investigator was provided with a sealed randomization code for each available allocation generated by an independent researcher. Both the patients and the investigator were blind to the study medication and the blindness was maintained by the similarity of the shape and color of both itraconazole and placebo capsules.

### 2.1. Inclusion Criteria

Eligibility for the study was restricted to female participants, aged 18 to 65 years old, and chemotherapy-naïve with a high functional capacity defined by an Eastern Cooperative Oncology Group Performance Status of zero or one. All individuals had histopathologically and radiologically confirmed stage III or IV ovarian epithelial malignancy, as staged by the eighth edition AJCC TNM classification system. Furthermore, entry mandated robust biochemical integrity, verified within 14 days of the initial chemotherapeutic cycle, encompassing sufficient hematological reserves (ANC > 1.5 × 10^9^/L; hemoglobin > 9 g/dL; platelets > 100,000/L), adequate liver function (total bilirubin ≤ 1.5 × ULN; AST/ALT ≤ 2 × ULN), and stable renal capacity (serum creatinine ≤ 1.5 × ULN or creatinine clearance > 50 mL/min).

### 2.2. Exclusion Criteria

The exclusion criteria included patients with a secondary primary malignancy; a history of hypersensitivity to paclitaxel, carboplatin, itraconazole, or chemically related azole compounds, and concurrent use of medications known to significantly affect itraconazole metabolism (e.g., anticonvulsants). Patients with hyperthyroidism which may increase itraconazole metabolism, were also excluded. Additional exclusion criteria comprised grade ≥ 2 peripheral neuropathy; uncontrolled concomitant medical conditions; active hepatitis or symptomatic hepatic disease; and historical or current evidence of uncontrolled ventricular dysfunction, including congestive heart failure or New York Heart Association (NYHA) class III or IV heart failure. Pregnant or breastfeeding women were also excluded.

### 2.3. Methods

#### 2.3.1. Demographic, Anthropometric, and Clinical Data of the Studied Patients

Age, weight, height, body mass index (BMI), body surface area (BSA), performance status were determined. Family history and medical history were collected. In addition, clinical examination, routine laboratory investigations, radiological staging of the ovarian tumor (MRI of abdomen and pelvis, CT abdomen, and pelvis (if MRI contraindicated)), trans-vaginal ultrasound, CT chest with contrast, bone scan, brain imaging (if symptomatic or clinically indicated), PET-CT when clinically indicated, and echocardiogram were performed.

#### 2.3.2. Blood Sampling and Quantification of Biological Markers

Five ml of venous blood samples were collected in the morning during the appointments that were scheduled for routine patient examinations before and after intervention. The collected blood samples were centrifuged at 3000 rpm for 15 min. The separated sera were stored at −80 °C till analysis of biological markers which were quantified using enzyme-linked immune-sorbent assay (ELISA) kits and included cancer antigen-125 “CA-125” (catalog no.: DL-CA125-Hu), vascular endothelial growth factor receptor-2 “VEGFR-2” (catalog no.: DLR-VEGFR2-Hu), and P-glycoprotein “P-gp” (catalog no.: DLR-Pgp-Hu).

#### 2.3.3. Clinical Assessment

The clinical outcome was assessed after the third and the sixth chemotherapy cycles and one year after completion of chemotherapy cycles using the Response Evaluation Criteria in Solid Tumors (RECIST), version 1.1 [11] through the assessment of tumor response. Overall response rate “ORR” is defined as “The sum of complete responses (CRs) with no detectable evidence of a tumor and partial responses (PRs) which is a decrease in tumor size” and disease control rate (DCR) is defined as “The percentage of patients with advanced cancer whose therapeutic intervention has led to a complete response, partial response, or stable disease”.

Furthermore, the quality of life (QOL) was assessed using the European Organization for Research and Treatment of Cancer (EORTC) QLQ-C30 version 3, QLQ-OV28 questionnaires [12].

#### 2.3.4. Assessment of Participants’ Adherence, Side Effects, and Tolerability

Participant adherence was assessed through counting the returned capsules. Subjects were submitted to close monitoring via weekly telephone communication and direct consultation during chemotherapeutic cycles to evaluate adherence and to record medication-associated adverse events. Adverse events were recorded and categorized according to National Cancer Institute Common Terminology Criteria for Adverse Events (NCI-CTCAE; version 5, 2017). Since paclitaxel is possibly associated with hepatotoxicity (which requires dose adjustment), liver functions were assessed through evaluation of ALT, AST, and bilirubin levels. Hepatotoxicity monitoring was based on evaluation of ALT, AST, and bilirubin levels together with patient symptoms. Hepatotoxicity is expected when there is an increase in bilirubin level greater than 1.5 mg/dL or when AST and ALT are greater than three times the normal range, in addition to patient symptoms which include abdominal pain, nausea or vomiting, fatigue, itchy skin, dark urine, and pale stool. Participants were considered non-adherent and excluded from the investigation if capsules remained unused or follow-up appointments were missed during any intervention period.

#### 2.3.5. The Primary and Secondary Endpoints

The principal outcome encompasses intergroup differences in overall response rate and disease control rate (RECIST). The secondary outcome involves alterations in the serum levels of biological markers (CA-125, VEGFR-2, P-gp).

#### 2.3.6. Sample Size Calculation

To ensure robust statistical power, a target recruitment of 66 participants was established, comprising 33 individuals per arm, which is a figure that accounts for an anticipated 10% attrition rate from a primary target sample of 60 in both arms. This sample size was strategically selected by modeling a prior randomized controlled trial that evaluated the impact of itraconazole in advanced non-small cell lung cancer. That pivotal study documented a significant increase in overall response rate (ORR) from 66.7% to 90% (*p* = 0.028) using an identical sample size. Ovarian and lung cancers are biologically distinct; therefore, the NSCLC trial was used solely as a pragmatic reference in the absence of prior ovarian cancer studies evaluating the efficacy of itraconazole. By anchoring the sample size estimation to these established efficacy data, the current study is adequately powered a priori to detect a clinically meaningful difference between the investigational arms.

#### 2.3.7. Statistical Analysis

All statistical analyses were executed using SPSS version 29 (IBM Inc., Chicago, IL, USA), employing a framework tailored to the specific nature of the data. The initial step involved assessing data distribution normality via the Shapiro–Wilks test. Parametric continuous variables were presented as mean ± standard deviation (SD) with pre-treatment versus post-treatment comparisons within a single arm analyzed using paired *t*-tests and between-group differences using unpaired *t*-tests. In contrast, non-parametric data are expressed as median with an interquartile range (IQR), and comparisons were performed using the Mann–Whitney U test. To analyze longitudinal data trajectories within each arm (pre-treatment, after three, and six chemotherapy cycles), a repeated measures ANOVA was implicated. Categorical variables, presented as frequencies and percentages, were analyzed with a chi-squared test or Fisher’s exact test as appropriate. Survival outcomes were visualized via the Kaplan–Meier method, and across all statistical evaluations, a two-sided *p*-value of less than or equal to 0.05 was considered the threshold for significance. Per-protocol analysis was applied in the present study; only data from patients who fully adhered to the study medications throughout the study course were involved in the final analysis.

## 3. Results

**Figure 1:**
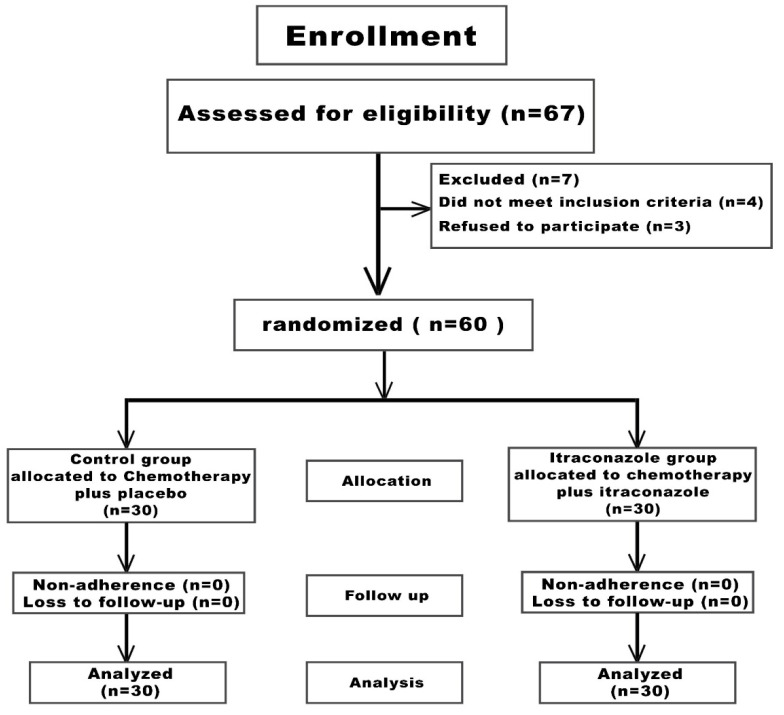
Study participants‘ flowchart.

Figure 1 depicts the participant flowchart. Among sixty-seven individuals evaluated for eligibility, seven participants were excluded (three individuals refused to participate and four participants failed to satisfy inclusion criteria). Consequently, the remaining 60 participants underwent randomization and allocation into the two study arms: arm 1 (control arm; n = 30) and arm 2 (Itraconazole arm; n = 30). Throughout the investigation period, neither group experienced loss of follow-up or demonstrated non-compliance. Thus, the allocated 60 participants (30 individuals per group) completed the investigation and their data were incorporated into the final analysis.

### 3.1. Demographic and Clinical Data of the Studied Patients

There is no significant variation between the two research groups regarding age, weight, height, BMI, BSA, family history, performance status, co-morbidity (hypertension, hypothyroidism, diabetes mellitus), stage according to TNM system, grade of tumor, and somatic BRCA mutation (*p* > 0.05). Also, there is no significant difference in surgical resection, neo-adjuvant chemotherapy, adjuvant chemotherapy, palliative therapy, number of chemotherapy cycles, and cumulative dose of Taxol or carboplatin (*p* > 0.05), as shown in Table 1.

### 3.2. Effect of Intervention on Clinical Response

Following the third and sixth chemotherapeutic cycles and relative to the control arm, the itraconazole arm demonstrated a significant enhancement in patient response (*p* = 0.035, *p* = 0.021, respectively) and overall response (*p* = 0.03, *p* = 0.015, respectively). Moreover, after the sixth chemotherapeutic cycle and as compared to the control arm, the itraconazole arm exhibited a significant improvement in disease control rate (*p* = 0.023), as illustrated in Table 2.

### 3.3. Effect of Itraconazole on the Assessed Biological Markers

At baseline, no significance variation between the two study arms was observed regarding serum concentrations of CA-125, VEGFR-2, and P-gp (*p*^2^ > 0.05) as shown in Table 3.

After intervention and as compared to baseline data, both study arms showed a significant decrease in CA-125 and P-gp serum concentrations (*p*^1^ < 0.001), which was associated with a significant elevation in VEGFR-2 serum level (*p*^1^ < 0.001), as shown in Table 3.

Following intervention, the itraconazole group demonstrated a significant reduction in serum CA-125 concentration after the sixth chemotherapy cycle as compared to the control group (*p*^2^ = 0.005), as presented in Table 3. Additionally, the itraconazole arm exhibited a significant decrease in serum P-gp concentration after the sixth chemotherapy cycle when contrasted with the control group (*p*^2^ = 0.042) as displayed in Table 3. Conversely, following the sixth chemotherapeutic cycle and relative to the control group, the itraconazole cohort revealed a significant increase in serum VEGFR-2 concentration (*p*^2^ = 0.006), as indicated in Table 3.

### 3.4. Effect of Intervention on Progression-Free Survival

The median follow-up duration for the overall study population was 17 months. The median follow-up duration was 18 months in the itraconazole group while it was 13.5 months in the placebo group. The median progression-free survival (PFS), which is defined as the time point at which 50% of patients experienced disease progression or death, was 13.5 months for the overall study population.

**Figure 2:**
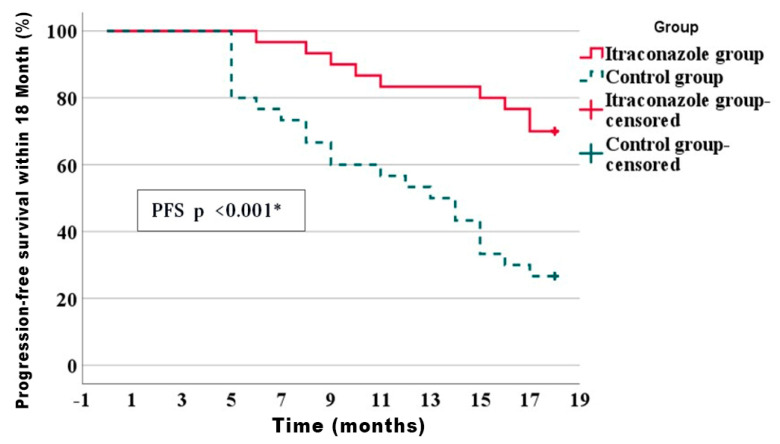
Kaplan–Meier of disease progression-free survival within 18 months of the studied groups. * Statistically significant difference.

Progression-free survival represents the mean duration following treatment initiation during which an individual remains alive without cancer growth or metastasis. Progression-free survival for participants who exhibited metastatic disease and who received palliative chemotherapy was evaluated 12 months after initiation of chemotherapy, and PFS was assessed at a fixed time point of 18 months following completion of chemotherapy for the overall study population. The comparative analysis between both study arms demonstrated significant improvements in progression-free survival within the itraconazole arm as compared to the control arm, which had twenty-one patients (70%) in the itraconazole arm versus eight patients (26.7%) in the control group (*p* ≤ 0.001) (Figure 2).

### 3.5. Effect of Intervention on Quality of Life (QOL)

The comparative analysis between both study groups regarding EORTC QLQ-C30 with a completion rate of 30/30 (100%) and Minimum Clinically Important Difference (MCID) thresholds ≥10-point change (likely clinically meaningful) demonstrated a significant improvement in global health status (*p* = 0.002), physical functioning (*p* = 0.009), role functioning (*p* = 0.033), emotional functioning (*p* = 0.011), cognitive functioning *(p* = 0.013), and social functioning (*p* = 0.023), in the favor of itraconazole group relative to the control group as illustrated in Table 4.

Treatment-related adverse effects were evaluated using patient questionnaires and revealed significant reduction in fatigue (*p* = 0.011), nausea and vomiting (*p* = 0.011), pain (*p =* 0.021), dyspnea (*p =* 0.030), insomnia (*p =* 0.003), appetite loss (*p =* 0.026), constipation (*p =* 0.001), and diarrhea (*p =* 0.009), in the favor of itraconazole group compared to the control group as demonstrated in Table 4.

EORTC QLQ-OV28 was administered to all participants with completion rate of 30/30 (100%) and Minimum Clinically Important Difference (MCID) thresholds ≥ 10-point change (likely clinically meaningful), whereby they demonstrated a significant reduction in gastrointestinal (*p =* 0.016), hormonal (*p =* 0.017), attitude (*p =* 0.047), and chemotherapy-associated adverse effects (*p =* 0.041), in the favor of itraconazole group compared to the control group as demonstrated in Table 5.

### 3.6. Drug Safety and Tolerability

There were no significant variations observed between the two study groups in the terms of reported adverse effect of the treatment including hematological toxicity such as anemia, neutropenia, thrombocytopenia, and non-hematological toxicity such as nausea, diarrhea, vomiting, peripheral neuropathy, cardiotoxicity, hepatotoxicity, and nephrotoxicity, (*p* > 0.05) as shown in Table 6.

## 4. Discussion

Notwithstanding chemotherapeutic advancements, five-year-free survival rates among individuals with ovarian cancer remain below 50%, which is primarily attributed to chemotherapeutic resistance. Both primary resistance (individuals demonstrate complete treatment unresponsiveness resulting in disease advancement) and secondary resistance (individuals subsequently develop resistance following initial therapeutic response) to platinum-based chemotherapy regimens correlate profoundly with unfavorable prognosis of individuals with epithelial ovarian cancer (EOC). For those individuals, comprehensive comprehension of resistance mechanisms represents a critical and unaddressed clinical priority [13].

Ovarian cancer cells exhibit intricate resistance mechanisms against chemotherapeutic agents through various interconnected processes including multidrug resistance (MDR), DNA damage repair (DDR), cellular metabolism, oxidative stress response, cell cycle control, cancer stem cells (CSCs), immune system interactions, apoptotic processes, autophagy mechanisms, and dysregulated signaling cascades. Consequently, no individual mechanism can comprehensively account for the treatment resistance observed in patients with ovarian cancer [5]. Multiple novel approaches are currently under investigation to address this resistance, including the combination of platinum-based chemotherapeutic regimens with emerging molecularly targeted therapeutics such as bevacizumab or Olaparib [5].

Itraconazole has shown antineoplastic properties through promoting cell cycle disruption, programmed cell death, and autophagy, while concurrently targeting numerous oncogenic pathways to suppress cell proliferation, metastasis, and invasive characteristics across diverse types of malignancy [7]. Chemotherapeutic resistance emergence among cancer patients constitutes a significant prognostic determinant. Itraconazole functions as an established p-glycoprotein antagonist capable of overcoming multidrug resistance mechanisms. Secondary to its potent antineoplastic properties and its favorable safety profile, itraconazole has undergone multiple clinical investigations which demonstrated substantial therapeutic efficacy [7]. This preceding evidence motivated the current investigation, which sought to assess the effect of itraconazole as an adjunctive treatment to paclitaxel and carboplatin on therapeutic response among individuals with late-stage epithelial ovarian malignancy.

The dose used itraconazole (400 mg orally) for 5 days for each chemotherapy cycle (2 days before chemotherapy, the day of chemotherapy, and 2 days after chemotherapy), which was based on some former studies, pre-clinical data, and safety considerations [10].

Throughout this investigation, after the third and sixth chemotherapy cycles, the itraconazole group demonstrated significantly enhanced therapeutic outcomes as compared to the placebo group whereas the proportion of participants who achieved complete and partial responses was significantly greater in the itraconazole group versus the placebo group. These previously mentioned findings appear to be consistent with observations from other researchers who examined the therapeutic role of itraconazole in patients with lung cancer and evaluated its impact on participant response [8]. Moreover, it was observed that the itraconazole group showed a significantly higher progression-free survival than the control group; this finding seems in consonance with a previous study which evaluated the effect of itraconazole on advanced non-small cell lung cancer [8]. These beneficial effects of itraconazole on patients’ response and progression-free survival could be attributed to the ability of itraconazole to inhibit various carcinogenic signaling pathways, such as hedgehog (Hh) and mammalian target of rapamycin (mTOR) pathways, and its capacity to induce apoptosis, autophagy, and cell cycle arrest [14].

Elevated serum CA-125 concentrations may be observed in patients with ovarian cancer; however, this biomarker demonstrates limited sensitivity during early-stage ovarian cancer [15]. Elevated CA-125 concentrations are additionally documented in various physiological and pathological states including menstrual cycles, gestation, endometriosis, and peritoneal inflammatory conditions [16]. Throughout the present investigation and following the third and sixth chemotherapeutic cycles, CA-125 concentrations demonstrated significant reduction in the itraconazole group as compared to the control group—a finding that aligns with the results derived from a previous study [17]. This favorable effect of itraconazole on CA-125 serum level could be justified on the basis that itraconazole could exert anti-tumor effects by inhibiting angiogenesis and hedgehog signaling pathways, both of which are implicated in ovarian cancer progression. By disrupting these pathways, itraconazole could reduce tumor burden and subsequently lower CA-125 levels. Additionally, itraconazole could enhance chemotherapy efficacy through increasing intertumoral drug concentrations which in turn might contribute to the observed decline in CA-125 level [18].

Vascular endothelial growth factor (VEGF) binds to vascular endothelial growth factor receptor (VEGFR) and plays an essential role in cancerous invasion and metastatic dissemination [19]. Recent studies have demonstrated that ovarian cancer cells display VEGFR and VEGF expression [20]. This indicates that VEGF can produce immediate impacts on ovarian cancer cells via autocrine and paracrine pathways [21]. Earlier investigations have proposed that VEGF participates in tumorigenesis, progression, invasion, and metastatic spread of ovarian malignancy [22]. Throughout the present investigation and particularly following the sixth chemotherapeutic cycle, human VEGFR-2 soluble protein concentration demonstrated a significant increase in the itraconazole arm relative to the control group. However, a previous study demonstrated that VEGFR-2 level was decreased following itraconazole treatment [9]. These conflicting findings may be attributable to differences in experimental methodologies including variations in dosing regimens, treatment duration, and cancer subtypes. Although itraconazole is known to inhibit VEGFR-2 phosphorylation, thereby blocking VEGF/VEGFR-2 binding, the observed increase in VEGFR-2 levels during the current study may attributed to a compensatory up-regulation adaptive response mediated by tumor cells.

The P-glycoprotein (P-gp, 170 kDa) molecule, encoded by the MDR-1 gene (specifically ABCB1), is a part of the ATP-binding cassette transporter family which facilitates intrinsic and acquired drug resistance pathways in malignant cells [23]. The elevated P-gp expression level correlates with reduced progression-free survival (PFS) duration in patients with epithelial ovarian cancer [24]. During our study and after the sixth chemotherapy cycle, the level of P-gp exhibited a significant decrease in the itraconazole group as compared to the control group. Our aforementioned finding is in agreement with the finding of other authors who proved the role of P-glycoprotein in tumor chemoresistance [25]. The effect of itraconazole on P-gp may be justified on the basis that itraconazole inhibits Wnt signal, which induces the senescence of dormant cells and mediates the reversal of multidrug resistance by P-glycoprotein [7].

Following the third and sixth chemotherapy cycles and when contrasted with the control arm, the itraconazole arm demonstrated a significant improvement across most dimensions of quality of life (QOL). This beneficial impact of itraconazole on QOL is attributed to the improvement in patients response since combining itraconazole with other chemotherapeutic drugs can produce better response with subsequent improvement in QOL [7].

During the current investigation, itraconazole was well-tolerated and demonstrated acceptable safety profile. The itraconazole group showed a statistically non-significant difference as compared to the control group concerning the documented side effects. This finding concerning the safety of itraconazole seems in agreement with some previous studies which revealed the safety of such drug [26].

The overall results obtained with the current study postulated that itraconazole was safe and its implication resulted in significant reductions in the serum levels of CA-125 and P-gp which indicate enhanced tumor shrinkage, reduced drug efflux activity, and greater tumor sensitivity to chemotherapy with subsequent improvement in clinical response which was translated by significant improvements in tumor response, overall response, disease control rate, and progression-free survival.

The strength points of the current work encompass its randomized placebo-controlled double-blind parallel design, and the implication of the same brand of itraconazole throughout the trial duration; to the best of our knowledge, this work may represent the inaugural clinical investigation directed at evaluating the efficacy of itraconazole in patients with ovarian cancer who received paclitaxel plus carboplatin chemotherapy. Nevertheless, this work has some constraints including the relatively small sample size (due to economic reason), being a single center study, the short follow-up duration, and the lack of stratified subgroups analysis in order to distinguish the treatment response of neo-adjuvant, adjuvant, and palliative patients. The lack of assessment of pharmacodynamic biomarkers (which allow mechanistic interpretation, establishment of proof-of-concept, and directly measures the response to treatment) and the lack of the implication of bevacizumab and Poly ADP-ribose polymerase (PARP) inhibitors (secondary to therapeutic unavailability) for patients enrolled in the study represent other limitations of the current work. Therefore, future multi-institutional and expanded investigations remain necessary.

## 5. Conclusions

This randomized placebo-controlled double-blind parallel study demonstrated the potential safety and effectiveness of itraconazole as an adjunct therapy to paclitaxel and carboplatin chemotherapy in improving therapeutic response and reducing chemotherapy resistance in patients with advanced epithelial ovarian cancer, which was translated by significant improvements in patients’ response, overall response rate, disease control rate, and progression-free survival, alongside significant declines in both CA-125 and P-glycoprotein serum concentrations. Notwithstanding these encouraging findings, future multi-institutional investigations remain necessary to validate these preliminary findings.

## 6. Future Directions

During the current study, itraconazole demonstrated potential effectiveness as an adjunct therapy to paclitaxel and carboplatin in improving therapeutic response and reducing chemotherapy resistance in patients with advanced epithelial ovarian cancer. However, its role as a maintenance therapy requires further investigation, particularly regarding pharmacologic interactions when combined with bevacizumab and Poly ADP-ribose polymerase (PARP) inhibitors. Furthermore, future phase IIb/III randomized trials are recommended to validate the clinical benefit observed in this preliminary study. These proposed future trials would use progression-free survival (PFS) as a primary endpoint, with CA-125, P-glycoprotein (P-gp), and angiogenic markers, such as VEGFR-2, and safety outcomes as key secondary endpoints. Furthermore, stratified subgroup analysis in order to distinguish the treatment response of neo-adjuvant, adjuvant, and palliative patients should be considered. Based on the effect sizes observed in the present study, the estimated sample size will be calculated with a statistical power of at least 80% at a two-sided α error = 0.05 to detect a clinically meaningful improvement in PFS. This design will enable more robust evaluation of itraconazole as an adjunct to standard chemotherapy in advanced epithelial ovarian cancer.

## Figures and Tables

**Table 1:** Demographic, anthropometric, and clinical data of the two study groups.

Variables		Control Arm (n = 30)	Itraconazole Arm (n = 30)	*p*-Value
Age (years)	Mean ± SD	54.7 ± 11.8	53.9 ± 12.4	0.799
	Range	21–64	20–65	
	≤60	15 (50%)	16 (53.3%)	0.796
	>60	15 (50%)	14 (46.7%)	
Weight (kg)	Mean ± SD	79.2 ± 13.4	84.9 ± 13.7	0.105
	Range	56–106	58–104	
Height (m)	Mean ± SD	1.64 ± 0.03	1.64 ± 0.04	0.943
	Range	1.57–1.69	1.56–1.71	
BMI (kg/m^2^)	Mean ± SD	29.48 ± 5.21	31.8 ± 5.8	0.119
	Range	20.2–39.9	20.55–41.14	
BSA (m^2^)	Mean ± SD	1.89 ± 0.17	1.95 ± 0.17	0.197
	Range	1.6–2.2	1.6–2.2	
Family history	Yes	4 (13.3%)	5 (16.7%)	1.00
	No	26 (86.7%)	25 (83.3%)	
Performance status	Grade 0	14 (46.7%)	11 (36.7%)	0.600
	Grade 1	16 (53.3%)	19 (63.3%)	
Hypertension		10 (33.3%)	10 (33.3%)	1.00
Diabetic		13 (43.3%)	13 (43.3%)	1.00
Hypothyroidism		1 (3.3%)	3 (10%)	0.612
T	T3	17 (56.7%)	17 (56.7%)	1.00
	T4	13 (43.3%)	13 (43.3%)	
N	Positive	14 (46.7%)	16 (53.3%)	0.796
	Negative	16 (53.3%)	14 (46.7%)	
M	Positive	12 (40%)	9 (30%)	0.588
	Negative	18 (60%)	21 (70%)	
Stage	Stage 3	18 (60%)	21 (70%)	0.588
	Stage 4	12 (40%)	9 (30%)	
Site of metastases	Peritoneal	7 (23.3%)	5 (16.7%)	0.871
	Liver	4 (13.3%)	3 (10%)	
	Other	1 (3.3%)	1 (3.3%)	
	No	18 (60%)	21 (70%)	
Histology of the tumor	Serous	30 (100%)	30 (100%)	---
Grade of the tumor	Grade 2	16 (53.3%)	17 (56.7%)	0.795
	Grade 3	14 (46.7%)	13 (43.7%)	
Somatic BRCA mutation	Yes	9 (30%)	6 (20%)	0.371
	No	21 (70%)	24 (80%)	
Surgical resection	Yes	19 (63.3%)	20 (66.7%)	0.786
	No	11 (36.7%)	10 (33.3%)	
Neo-adjuvant chemotherapy	Yes	10 (33.3%)	12 (40%)	0.788
	No	20 (66.7%)	18 (60%)	
Adjuvant chemotherapy	Yes	13 (43.3%)	11 (36.7%)	0.792
	No	17 (56.7%)	19 (63.3%)	
Palliative	Yes	11 (36.7%)	10 (33.3%)	0.788
	No	19 (63.3%)	20 (66.7%)	
Number of chemotherapy cycles	6 cycles	30 (100%)	30 (100%)	---
Cumulative dose of Taxol (mg)	Mean ± SD	2041.2 ± 184.6	2102.4 ± 178.8	0.197
	Range	1728–2376	1728–2376	
Cumulative dose of carboplatin (mg)	Mean ± SD	3281.67 ± 528.09	3063.33 ± 481.72	0.100
	Range	2700–4800	2700–4800	

Data expressed as mean ± standard deviation, range number, and percentage (%). m: meter; BMI: body mass index; kg: kilogram; m^2^: meter square; BSA: body surface area; T: tumor size; N: number of lymph node involvement; M: metastases; BRCA: breast cancer gene; mg: milligram. Significance level was set at *p* ≤ 0.05. *p*-value: difference between the two groups.

**Table 2:** Response, ORR, and DCR after the third and the sixth chemotherapy cycle for the two study groups.

			Control Arm (n = 30)	Itraconazole Arm (n = 30)	*p*-Value
Response After the third chemotherapy cycle	CR		3 (10%)	8 (26.7%)	0.035 *
	PR		12 (40%)	16 (53.3%)	
	SD		15 (50%)	6 (20%)	
	DP		0 (0%)	0 (0%)	
ORR			15 (50%)	24 (80%)	0.030 *
DCR			30 (100%)	30 (100%)	**--**
Response After the sixth chemotherapy cycle		CR	6 (20%)	11 (36.7%)	0.021 *
		PR	8 (26.7%)	13 (43.3%)	
		SD	10 (33.3%)	6 (20%)	
		DP	6 (20%)	0 (0%)	
ORR			14 (46.7%)	24 (80%)	0.015 *
DCR			24 (80%)	30 (100%)	0.023 *

Data expressed as number and percentage (%). CR: complete response; PR: partial response; SD: stationary disease; DP: disease progression; ORR: overall response rate; DCR: disease control rate. Significance level was set at *p* ≤ 0.05. *p*-value: difference between the two groups in treatment response, overall response rate, and disease control rate. * Statistically significant difference. Bold formatting is used to denote statistically significant results.

**Table 3:** The assessed biological markers for the two study groups.

Biomarkers		Control Arm (n = 30)	Itraconazole Arm (n = 30)	*p*^2^-Value
CA-125	Baseline	433 (266.25–659.25)	367 (44.5–481)	0.098
	After the sixth chemotherapy cycle	304 (166–484)	84 (36.3–272.5)	0.005 *
	*p* ^1^	<0.001 *	<0.001 *	
VEGFR-2	Baseline	5.41 ± 0.91	5.77 ± 1.01	0.152
	After the sixth chemotherapy cycle	6.59 ± 0.92	7.3 ± 0.9	0.006 *
	*p* ^1^	<0.001 *	<0.001 *	
P-gp	Baseline	4.4 ± 0.5	4.37 ± 0.51	0.899
	After the sixth chemotherapy cycle	3.46 ± 0.66	3.12 ± 0.64	0.042 *
	*p* ^1^	<0.001 *	<0.001 *	

Data expressed as median (IQR), mean ± SD. CA-125: cancer antigen 125; VEGFR-2: vascular endothelial growth factor receptor type 2; P-gp: P-glycoprotein. Significance level was set at *p* ≤ 0.05. *p*^1^-value: probability of significance within the same group (baseline versus after the sixth chemotherapy cycle data); *p*^2^-value: probability of significance between the two study groups. * Statistically significant difference. Bold formatting is used to denote statistically significant results.

**Table 4:** EORTC QLQ-C30 for the two study groups.

	Control Arm (n = 30) Mean ± SD	Itraconazole Arm (n = 30) Mean ± SD	*p*-Value
**Global health status**			
Baseline	68.8 ± 5.9	67.7 ± 6.1	0.467
After the third chemotherapy cycle	77.7 ± 5.2	81.5 ± 5.5	0.013 *
After the sixth chemotherapy cycle	82.7 ± 5.1	85.4 ± 4.7	0.002 *
**Physical functioning**			
Baseline	76.8 ± 4.8	75.8 ± 4.7	0.451
After the third chemotherapy cycle	83.6 ± 4.2	85.8 ± 3.4	0.034 *
After the sixth chemotherapy cycle	92.1 ± 3.1	94.0 ± 1.5	0.009 *
**Role functioning**			
Baseline	82.1 ± 2.5	82 ± 2.4	0.834
After the third chemotherapy cycle	92.8 ± 1.5	93.6 ± 1.7	0.007 *
After the sixth chemotherapy cycle	94.8 ± 0.9	95.5 ± 1.3	0.033 *
**Emotional functioning**			
Baseline	66.5 ± 4.7	67.3 ± 4.4	0.464
After the third chemotherapy cycle	81.3 ± 5.0	84.2 ± 4.5	0.020 *
After the sixth chemotherapy cycle	84.6 ± 4.9	87.9 ± 4.6	0.011 *
**Cognitive functioning**			
Baseline	62.2 ± 3.2	62.7 ± 3.5	0.622
After the third chemotherapy cycle	76.7 ± 3.3	78.8 ± 3.5	0.025 *
After the sixth chemotherapy cycle	80.0 ± 3.4	82.3 ± 3.5	0.013 *
**Social functioning**			
Baseline	80.4 ± 2.3	80.5 ± 2.6	0.875
After the third chemotherapy cycle	94.5 ± 2.1	96.4 ± 2.2	0.001 *
After the sixth chemotherapy cycle	96.43 ± 1.3	97.4 ± 1.7	0.023 *
**Fatigue**			
Baseline	43.4 ± 2.6	43.2 ± 2.8	0.739
After the third chemotherapy cycle	29.9 ± 2.8	28.6 ± 2.5	0.055
After the sixth chemotherapy cycle	26.6 ± 2.8	24.7 ± 2.9	0.011 *
**Nausea and vomiting**			
Baseline	12.4 ± 2.8	12.8 ± 2.6	0.531
After the third chemotherapy cycle	5.9 ± 2.0	4.5 ± 1.9	0.008 *
After the sixth chemotherapy cycle	3.8 ± 2.1	2.5 ± 1.9	0.011 *
**Pain**			
Baseline	29.2 ± 2.3	28.8 ± 2.7	0.474
After the third chemotherapy cycle	21.2 ± 2.0	19.8 ± 2.3	0.017 *
After the sixth chemotherapy cycle	17.2 ± 2.0	15.8 ± 2.7	0.021 *
**Dyspnea**			
Baseline	10.6 ± 2.2	10.0 ± 2.2	0.323
After the third chemotherapy cycle	4.8 ± 1.7	3.7 ± 1.8	0.015 *
After the sixth chemotherapy cycle	2.8 ± 1.9	1.9 ± 1.1	0.030 *
**Insomnia**			
Baseline	10.8 ± 1.7	10.6 ± 1.7	0.644
After the third chemotherapy cycle	5.7 ± 1.5	4.5 ± 1.2	0.001 *
After the sixth chemotherapy cycle	4.3 ± 1.5	3.1 ± 1.4	0.003 *
**Appetite loss**			
Baseline	7.6 ± 1.9	7.0 ± 1.9	0.240
After the third chemotherapy cycle	6.1 ± 1.5	6.3 ± 1.9	0.666
After the sixth chemotherapy cycle	4.2 ± 1.9	3.1 ± 1.7	0.026 *
**Constipation**			
Baseline	12.2 ± 1.8	11.6 ± 2.3	0.274
After the third chemotherapy cycle	7.2 ± 1.5	5.8 ± 1.8	0.002 *
After the sixth chemotherapy cycle	4.2 ± 1.7	2.8 ± 1.5	0.001 *
**Diarrhea**			
Baseline	5.5 ± 1.8	5.8 ± 1.6	0.493
After the third chemotherapy cycle	2.1 ± 0.8	1.6 ± 0.9	0.023 *
After the sixth chemotherapy cycle	1.1 ± 0.7	0.6 ± 0.6	0.009 *
**Financial**			
Baseline	11.4 ± 2.0	10.9 ± 1.9	0.409
After the third chemotherapy cycle	5.8 ± 1.8	5.9 ± 1.4	0.871
After the sixth chemotherapy cycle	3.7 ± 1.8	4.1 ± 1.6	0.333

Data expressed as mean ± SD. EORTC: European Organization for Research and Treatment of Cancer. QLQ-C30: quality of life questionnaire contains 30 questions. Significance level was set at *p* ≤ 0.05. * Statistically significant difference. Bold formatting is used to denote statistically significant results.

**Table 5:** EORTC QLQ-OV28 questionnaire for the two study groups.

	Control Arm (n = 30) Mean ±SD	Itraconazole Arm (n = 30) Mean ± SD	*p*-Value
**Gastrointestinal tract disorder**			
Baseline	41.6 ± 2.5	42.4 ± 2.3	0.189
After the third chemotherapy cycle	41.8 ± 3	40.3 ± 2.4	0.029 *
After the sixth chemotherapy cycle	38.4 ± 2.8	36.7 ± 2.4	0.016 *
**Peripheral neuropathy**			
Baseline	26.7 ± 3.4	27.7 ± 3.2	0.276
After the third chemotherapy cycle	34.5 ± 3.6	33.0 ± 3.3	0.101
After the sixth chemotherapy cycle	39.0 ± 3.0	37.5 ± 3.6	0.077
**Hormonal**			
Baseline	11.3 ± 2.1	11.1 ± 2.2	0.767
After the third chemotherapy cycle	18.8 ± 2.6	17.0 ± 2.9	0.016 *
After the sixth chemotherapy cycle	23.6 ± 2.7	21.6 ± 3.4	0.017 *
**Body image**			
Baseline	10.0 ± 2.2	10.6 ± 2.2	0.323
After the third chemotherapy cycle	2.6 ± 2.0	3.2 ± 1.7	0.279
After the sixth chemotherapy cycle	1.5 ± 1.0	2.3 ± 1.9	0.078
**Attitude**			
Baseline	51.6 ± 3.0	52.1 ± 3.8	0.575
After the third chemotherapy cycle	73.5 ± 8.8	69.5 ± 4.9	0.035 *
After the sixth chemotherapy cycle	78.8 ± 8.9	74.9 ± 5.1	0.047 *
**Chemotherapy side effects**			
Baseline	13.6 ± 2.8	13.1 ± 3.3	0.560
After the third chemotherapy cycle	20.0 ± 3.3	18.0 ± 3.5	0.030 *
After the sixth chemotherapy cycle	24.9 ± 3.2	23.2 ± 3.4	0.041 *
**Sexuality**			
Baseline	8.5 ± 1.7	8.4 ± 1.7	0.884
After the third chemotherapy cycle	5.6 ± 1.8	6.0 ± 2.0	0.330
After the sixth chemotherapy cycle	3.0 ± 1.9	3.9 ± 1.9	0.314

Data expressed as mean ± SD. EORTC: European Organization for Research and Treatment of Cancer. QLQ-OV28: quality of life questionnaire—ovarian cancer contains 28 questions. Significance level was set at *p* ≤ 0.05. * Statistically significant difference. Bold formatting is used to denote statistically significant results.

**Table 6:** The reported adverse events for the two study groups.

Adverse Effects		Control Group (n = 30)	Itraconazole Group (n = 30)	*p*-Value
Anemia	G1 and 2	26 (86.7%)	27 (90%)	1.00
	G3 and 4	4 (13.3%)	3 (10%)	
Neutropenia	G1 and 2	15 (50%)	18 (60%)	0.436
	G3 and 4	15 (50%)	12 (40%)	
Thrombocytopenia	G1 and 2	27 (90%)	26 (86.7%)	1.00
	G3 and 4	3 (10%)	4 (13.3%)	
Nausea	G1 and 2	26 (86.7%)	24 (80%)	0.730
	G3 and 4	4 (13.3%)	6 (20%)	
Diarrhea	G1 and 2	6 (20%)	12 (40%)	0.159
	No	24 (80%)	18 (60%)	
Vomiting	G1 and 2	28 (93.3%)	29 (96.7%)	1.00
	G3 and 4	2 (6.7%)	1 (3.3%)	
Peripheral neuropathy	G1 and 2	18 (60%)	17 (56.7%)	0.793
	No	12 (40%)	13 (43.3%)	
Hepatotoxicity	G1 and 2	7 (23.3%)	5 (16.7%)	0.748
	No	23 (76.7%)	25 (83.3%)	
Nephrotoxicity	G1 and 2	5 (16.7%)	3 (10%)	0.706
	No	25 (83.3%)	27 (90%)	
Cardiotoxicity	G1 and 2	0 (0%)	2 (6.7%)	---
	No	30 (100%)	28 (93.3%)	

Data expressed as number and percentage (%). Graph 1. grade 1 adverse effects. Graph 2. grade 2 adverse effects. Graph 3. grade 3 adverse effects. Graph 4. grade 4 adverse effects. Significance level was set at *p* ≤ 0.05.

## Data Availability

Data can be obtained upon reasonable request from the corresponding author.
